# A smart pill bottle and text messaging intervention for promoting medication adherence in patients with chronic myeloid leukemia: a pilot study of *txt4 TKI*

**DOI:** 10.1007/s00520-025-09537-z

**Published:** 2025-05-24

**Authors:** Kuang-Yi Wen, Neil Palmisiano, Rita Smith, Rachel Slamon, Gina Keiffer, Christian Fidler, Margaret Kasner, Lindsay Wilde

**Affiliations:** 1 Department of Medical Oncology, Thomas Jefferson University, Philadelphia, PA 19107 USA; 2 Rutgers Cancer Institute, Rutgers University, New Brunswick, NJ USA; 3 Department of Medical Oncology, Jefferson Abington Hospital, Willow Grove, PA USA

**Keywords:** CML, TKI adherence, MHealth behavioral interventions, Supportive care

## Abstract

**Background:**

Patients undergoing tyrosine kinase inhibitor (TKI) therapy for chronic myeloid leukemia (CML) often face challenges with adherence, despite the efficacy of the treatment. Theory-guided and evidence-informed interventions addressing psychosocial and adherence barriers are critical for promoting adherence. Using a mixed-method approach, the pilot study developed and evaluated the feasibility and potential impact of an intervention “txt4TKI,” to support medication and symptom management among CML patients undergoing TKI therapy.

**Methods:**

Guided by the Necessity and Concerns framework, the study comprised two phases: Phase 1 involved qualitative patient needs assessment interviews informing intervention design. Phase 2 was a single-arm pilot study conducted for 6 months. The objectives of Phase 2 were to assess the feasibility and acceptability of the *txt4 TKI* intervention, examining enrollment and retention rates, intervention utilization, patient satisfaction, and changes in TKI medication adherence, psychosocial and symptom factors, from baseline to the 6-month follow-up. The intervention integrates a smart pill bottle activated with light, chimes to remind medication taking and three proactive text messages per week addressing clinical importance of TKI, symptom, lifestyle, and distress management as well as a biweekly toxicity assessment with tailored management feedback. Adherence was also objectively measured by the smart pill bottle and psychosocial survey was repeatedly assessed at 3- and 6-month.

**Results:**

The intervention, developed iteratively and theoretically through patient-centered inputs (*n* = 10) and evidence-based information during Phase 1, integrates knowledge on CML, TKI therapy, self-care symptom management, emotional support, and healthy lifestyle recommendations. In Phase 2, 20 out of 30 eligible patients (67% consent rate), with a mean age of 55.3, 60% females and 65% Non-White participants, were enrolled. Fifty-five percent have been taking their medication for 1–3 years and 50% were taking Dasatinib. A high satisfaction and engagement rate of 90% with an 85% retention rate were observed. While medication self-efficacy significantly improved from baseline to 6 months, the adherence rate to TKI was slightly decreased over time. Post-intervention interviews indicated that participants found the intervention user-friendly, providing valuable information and emotional support, but adjusting the timing and content of interventions to tailor to different stages of therapy might better support sustained adherence.

**Conclusions:**

The *txt4 TKI* intervention using interactive text messaging and a smart pill bottle, feasible, and acceptable, demonstrated an increase in medication self-efficacy, an important aspect of medication adherence behaviors. Further research is needed to explore factors that influence the promotion and maintenance of adherence behaviors.

## Introduction

Survival rates in patients with chronic myeloid leukemia (CML) significantly increased with the advent of the first tyrosine kinase inhibitor (TKI), imatinib [1]. Currently, imatinib, nilotinib, dasatinib, bosutinib, ponatinib, and asciminib stand as standard-of-care oral TKI therapies for CML, demonstrating notable success in enhancing progression-free and overall survival [2]. Despite this progress, the need for continuous and daily TKI intake poses challenges in maintaining high adherence, with studies indicating suboptimal adherence levels in a substantial portion of CML patients [3, 4].

Non-adherence to TKI has been associated with compromised treatment responses and escalated healthcare costs [5–9]. The side effects of TKIs, including myelosuppression, nausea, diarrhea, fatigue, myalgias, rash, and edema, contribute to patients discontinuing medication [10, 11], with adverse events and patient-reported symptoms correlating with lower adherence levels [3, 12]. Addressing these factors is crucial for ensuring optimal treatment outcomes. While forgetfulness remains a common reason for unintentional nonadherence, there is a limited understanding of effective patient-friendly interventions to promote TKI adherence [13].

Mobile health (mHealth) interventions, particularly text messaging (TXT) and Chatbots, present promising avenues for improving adherence. TXT-based interventions have shown success in various medical contexts, including breast cancer therapy adherence [14, 15], while Chatbots offer accessibility and structured interactions, especially for older cancer patients [16]. While electronic patient-reported outcome (ePRO) systems have demonstrated benefits in various health aspects [17–19], their impact on CML patients remains underexplored. Only one mHealth study targeting CML patients was identified [20], a 10-week pilot that included daily medication reminders, routine side effect assessments, tailored feedback via a web platform and text messages, and nurse consultations. While the study was deemed feasible with a small sample of 10 participants, it did not include objective measures of medication adherence and was limited by its short duration. Traditional measures of oral medication adherence, such as self-report or clinic pill counts, may not capture the complexity of patient behavior. Emerging technologies, like smart pill bottles, offer objective assessment by tracking medication timing. These smart pill bottles also show promise in promoting adherence through reminders and communication with patients, as evidenced in studies with HIV [21] and heart failure patients [22]. Currently there is a gap in understanding the potential benefits of such technologies in optimizing medication adherence for CML patients.

In this study, we aimed to develop and assess the feasibility and acceptability of a mobile adherence-promoting intervention, named *txt4 TKI*. This intervention integrates a smart pill bottle, interactive TXT, and a Chatbot for toxicity assessment with tailored education support for promoting adherence to TKI and symptom management. By combining these elements, we aimed to provide a user-friendly and engaging program for CML patients undergoing TKI therapy through offering proactive support, real-time tailored self-care advice, and facilitating adherence monitoring. The study aimed to fill existing gaps in examining the potential of digital technologies to enhance CML patient outcomes in this context.

## Methods

### Study aim and design

The pilot study aimed to develop and test *txt4 TKI*, a mHealth intervention for CML patients undergoing TKI therapy. Employing a mixed methods design, the study comprised two main phases: Phase 1 involved qualitative interviews informing intervention design, while Phase 2 was a single-arm pilot study conducted over six months. The objectives of Phase 2 were to assess the feasibility and acceptability of the *txt4 TKI* intervention, examining enrollment and retention rates, intervention utilization, patient satisfaction, and changes in TKI medication adherence, psychosocial factors, and symptom distress from baseline to the 6-month follow-up.

### Compliance with ethnical standards

The study was granted approval by Thomas Jefferson University’s institutional review board and the Sidney Kimmel Comprehensive Cancer Center’s ethics committee. Prior to participation in the study, written informed consent was acquired from all participants. Careful steps were taken to ensure the anonymity of all collected data, including interview transcripts, survey responses, and data obtained through text messages or the chatbot interface. For text message exchanges, we used end-to-end encryption, ensuring that messages remained private between patients and the system. Similarly, chatbot interactions are designed to be secure, with patient data being de-identified to prevent any traceability. These measures collectively improve the privacy and security of patient interactions within the intervention, aligning with the ethical standards and regulations governing the protection of patient information. The study strictly adhered to national and international regulations pertaining to the safeguarding of personal information and privacy, as necessitated by the use of digital technology.

### Phase 1 Patient qualitative interviews

In Phase 1 of our study, we systematically reviewed existing literature and evidence-based guidelines to inform the development of self-care message tips. An alert algorithm based on PRO-CTCAE was devised to guide patients on when to seek clinician advice or emergency care. Additionally, coping strategies for symptom management were integrated into the algorithm. To ensure a patient-centered approach, we conducted interviews with 10 CML patients, who were either currently or previously on TKI therapy. The sample included a diverse group comprising 7 females and 3 males, aged 20 to 72, with diagnoses spanning from 2 months to 9 years. They were prescribed imatinib (*n* = 4), dasatinib (*n* = 5), or nilotinib (*n* = 1).

We employed remote semi-structured interviews, utilizing concept elicitation techniques to delve into the patients'perspectives on their adherence to therapy, and overall disease management. The interview guide included key questions: 1) Could you please share your experience with taking the medicine for CML?; 2) What challenges or difficulties have you encountered while taking this medicine?; 3)How do you cope with these challenges?; 4) What are your opinions or concerns about this medicine?; and 5) In your view, what other strategies do you think would be helpful?

Interviews were transcribed verbatim and analyzed using Nvivo, employing applied thematic analysis methods and framed by the Necessity-Concerns Framework (NCF) [23] and generated the themes below. Corresponding patient quotes for each theme are listed in Table 1.

**Table 1:** Patient interview quote samples guided by the necessity-concerns framework

Perceived Necessity for TKI	Drivers for survival	"You want to see the bad cells, the cancer cells decrease. You want to live, you want to be here, and if you have a will to be here, then you want to take your medication and deal with the side effects" "I dread each meal in the evening because I take them at dinner time. I dread taking the pills still to this day. Um, but I have no choice."
	Benefits on overall health	"I knew I had to take this to keep my numbers down because when my numbers got up, I got more sick, but once I started taking it, my count went down." "Then when I was put on medicine, I realized after a year of taking the medicine, I hadn't had pneumonia, so I feel as though that medicine is actually, you know, helping me not get sick."
Concerns about TKI and Communication with Oncologists	Side effect challenges	“I have some neuropathy.., I can't even say it's just the GI issues, but that's a primary. But again, the swelling, facial swelling, the eyes, that's pretty horrible too. I feel like as soon as I started taking them, my eyes swelled up my face, swells up” “I'm just always so tired. I don't go out too much I try to find the energy sometimes to do other things, but it's difficult. that's the biggest lifestyle change of mine that not doing too much and I worked part-time now since I've been diagnosed, just because of fatigue” “Side effects are almost every day. The constant diarrhea, vomiting, maybe once a month, tiredness and fatigue, more often. This one kind of puts me in a depression mode. And, my stomach will hurt sometimes”
	Under-reported side effects due to challenges in doctor-patient communication	"I feel like I have to constantly go back to my primary doctor for my side effects because my oncologist doesn't usually feel like they're related. I had pain in my hip, and they said, okay, go see your primary care doctor. And you know, nobody wants to prescribe anything. So you just deal with the pain." "So some of the side effects, I just had to stop calling the doctor about. When I have an appointment, I'll mention them, but they don't seem too interested in talking about them or asking about them or doing anything about it."
Non-Adherence Factors and Medication Management	Intentional and Nonintentional Adherence Challenges	“We were going away and I did not want to being so tired all the time. So I stopped taking the tablets for a little bit..I did not tell my doctor” “Sometimes remembering whether or not I took the medicine is also difficult. In the morning. I'll think twice about whether or not actually. if I don't remember whether or not I took it I just wait till the next day. “ “I'm visiting my family right now. And like we went out to a park a few days ago and we were leaving around 9:30 in the morning. And, um, I forgot to take my pills with me”
	Coping Strategies	“So I use the app safe, um, that has just a daily push notification that comes on your phone screen. I started with that popping up around 1730 in the morning for me.” “So I always have at least like eight in my wallet and I always have the, the pill, extra say keep that beside my bed. So there's never a reason not to.” “I would say family members. I had to take my pill at 3:00 PM every day. So literally at 2:58 mom and then 2:49 dad called, and then grandmother at three o'clock and then 3:30, call again: are you sure you took it? that help..I already knew my, my family loved me” “As soon as I take them, I have a GI issues, so I know I have to take them to dinner when I'm at home, for me, I need to take them at my latest meal at home because I can sleep a lot of the side effects away”

### Perceived necessity for TKI

The participants underscored their commitment to TKI based on their understanding of its life-saving importance. Driven by a strong desire for survival, oral TKI treatment was often perceived as the last line of defense, especially for those who were not eligible for surgical options. For some patients, the initiation of oral TKI marked a shift from curative to palliative care, highlighting the acknowledgment of the disease’s non-curable nature. Despite coping with side effects, an occurring theme was a resilient determination to take on the challenges triggered by treatment. Many participants also emphasized that adhering to TKI served to mitigate illness. These perspectives underscore the perceived positive impact and beliefs of TKI on outcomes.

### Concerns about TKI and communication with oncologists

Side effects from TKI treatment were quite common, reported as both physical and emotional challenges. Everyday physical symptoms like nausea, pain, skin problems, and tiredness were commonly mentioned. Participants shared that managing these disease’s and treatment’s difficulties had a noticeable impact on their daily routines including their adherence behaviors to their treatment plan. Furthermore, some patients expressed hesitancy in communicating with their oncologists because they were reluctant to taking additional medications, or assumed they could manage independently, or perceived oncologists as either too busy or unresponsive to their concerns.

### Non-adherence factors and medication management strategies

Some patients occasionally decided to skip their TKI medication intentionally, to avoid side effects. This allowed them, for example, to eat and drink normally on social/religious occasions or during periods of illness, which could be further complicated by medication that involved fasting prior to administration. Unintentional non-adherence was also reported, commonly due to simple forgetfulness or caused by a change in routine, such as travelling or social occasions. Patients also developed certain strategies to enhance their TKI medication adherence, for example, planning routines and receiving support from their families. Strategies extended to coping symptoms and side effects, such as taking medication around meals or bedtime to alleviate discomfort.

The insights learned from these interviews highlight the multifaceted needs of individuals undergoing TKI therapy for CML. These findings emphasize the significance of addressing not only physical symptoms but also emotional well-being and the provision of knowledge and strategies to empower patients throughout their medication adherence management process. Qualitatively understanding these aspects helped us to develop the *txt4 TKI* intervention that addresses the needs of patients with CML undergoing TKI therapy.

### Phase 2 Pilot intervention study

#### Overview

**Fig. 1:**
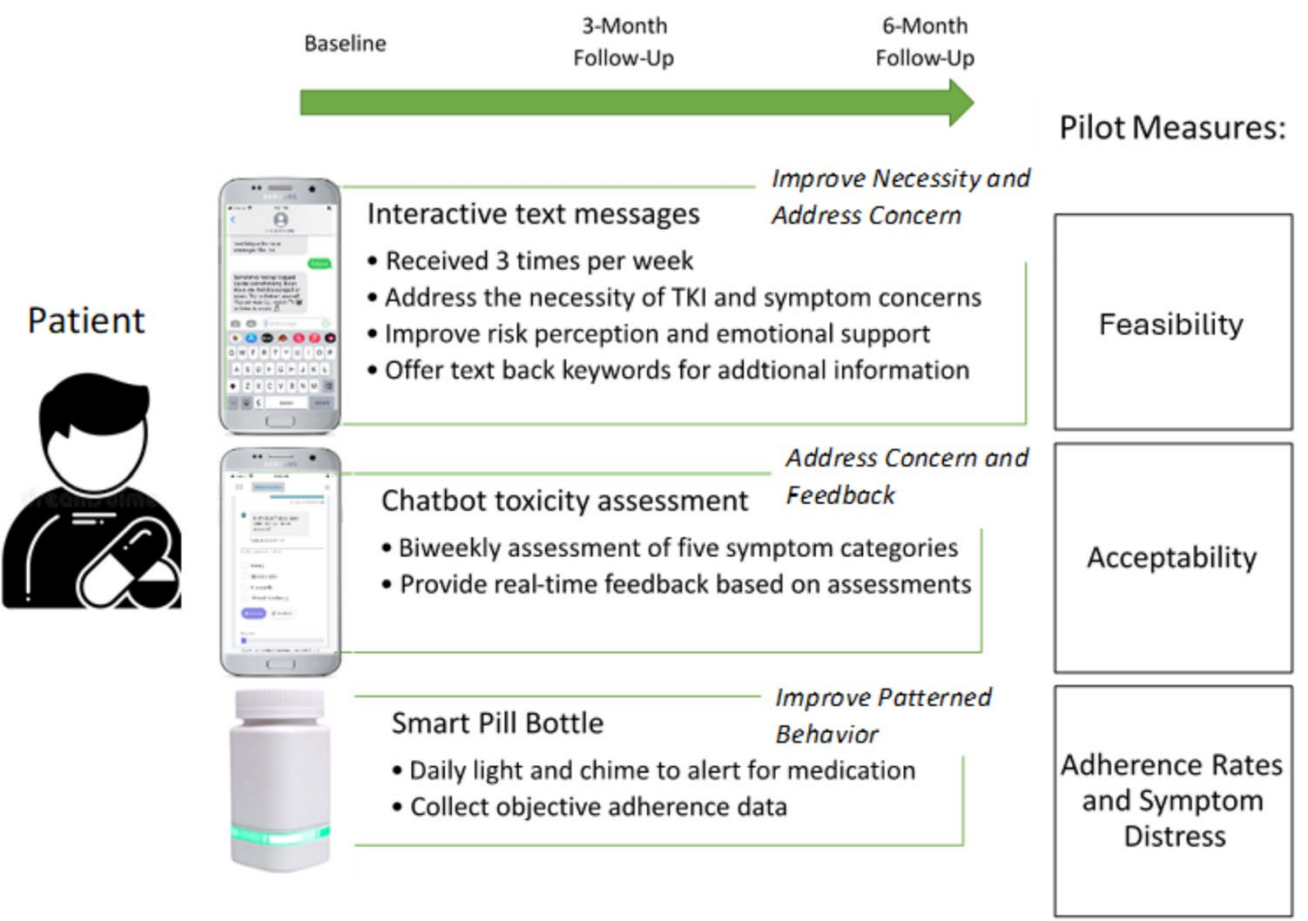
*txt4 TKI* intervention design and measures

The aim of phase 2 was to conduct a 6-month pilot test of the *txt4 TKI* intervention among patients with CML undergoing TKI therapy. This phase aimed to assess the intervention’s feasibility, as reported by accrual and attrition rates and the intervention’s acceptability by measuring patient satisfaction ratings and conducting postintervention interviews. Participant-reported psychosocial outcomes were also repeatedly evaluated as well as objective adherence rates measured by the smart pill bottle. Figure 1 demonstrates the pilot intervention design and measures, guided by the Necessity and Concerns Framework for medication adherence.

### The txt4 TKI intervention

**Fig. 2:**
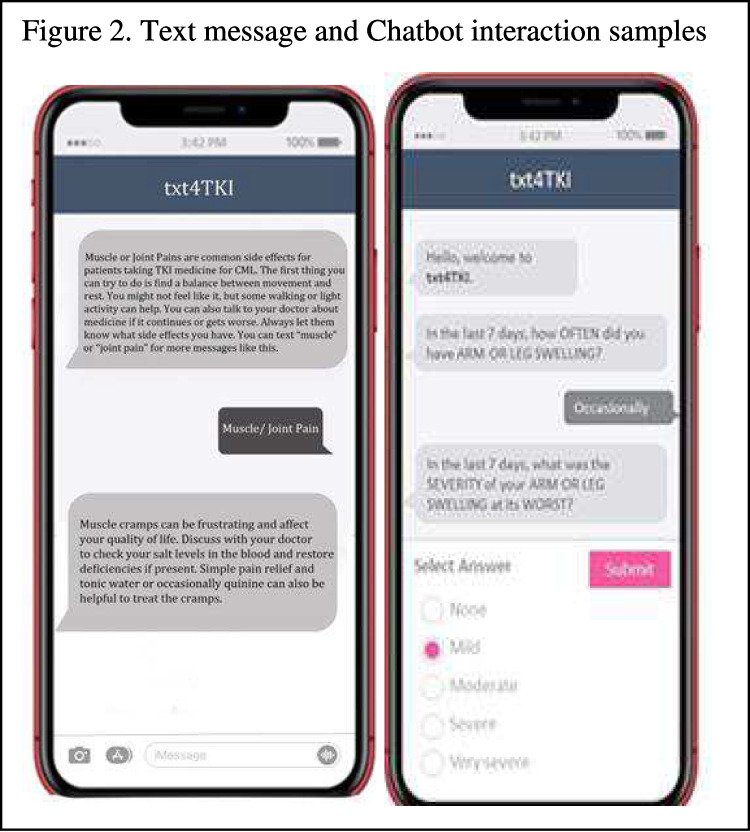
Text message and Chatbot interaction samples

Based on the findings from our phase 1 interviews, a review of evidence-based resources and the Necessity-Concerns Framework (NCF)[23], the *txt4 TKI* intervention was designed with three functions:

1) Proactive interactive text messages sent three times a week addressing knowledge of CML and TKI, side-effect management, self-care lifestyle behavioral change suggestions, emotional support and validation, and tips for communication with providers, patients were encouraged to text back using the predefined topic keywords to request additional desire information; see Fig. 2 for sample text messages.

2) A Chatbot symptom monitoring with tailored self-care feedback interface sent via texts to monitor symptoms experienced on a biweekly basis. This feature allowed participants to assess their symptoms at their convenience, providing them with a user-friendly and accessible method of monitoring their experiences. An integral aspect of this intervention component was the immediate feedback based on the symptom assessments collected via the Chatbot. This feedback offered participants self-care guidance in response to their reported symptoms. This timely and tailored support aimed to empower patients with actionable steps to manage their symptoms. See Fig. 2 for sample Chatbot interface interaction.

The Chatbot symptom checker application is a semi-automatized messaging system, delivered via TXT on smartphones. Severity and frequency assessment was adapted from the PRO-CTCAE (Patient-Reported Outcomes version of the Common Terminology Criteria for Adverse Events), a list of short consecutive questions was sent to patients biweekly to track the prevalence and severity of selected symptoms including nausea, diarrhea, muscle or joint pain, rash and swelling, relevant during TKI, identified by medical oncologists on the team. The Chabot then would tailor education and symptom management tips through the interactive question flow and answer exchanges. The algorithms were consistent with the CTCAE’s grade definitions: a) tailored self-care advice and access to evidence-based resources was provided for reported grade 1 or grade 2 symptom; b) a grade 3 reported symptom would suggest participants to contact their healthcare team; and c) a grade 4 reported symptom would advise participants to go to their closest ER for immediate assistance. Participants were reminded that the information we have collected was not shared with their providers and the advice provided should not be used as a replacement for medical advices and appointments. Our decision to use a TXT-delivered app over a native app was primarily due to accessibility and cost factors. The symptom checker tool is web-based and therefore not stored on a user’s device.

**Fig. 3:**
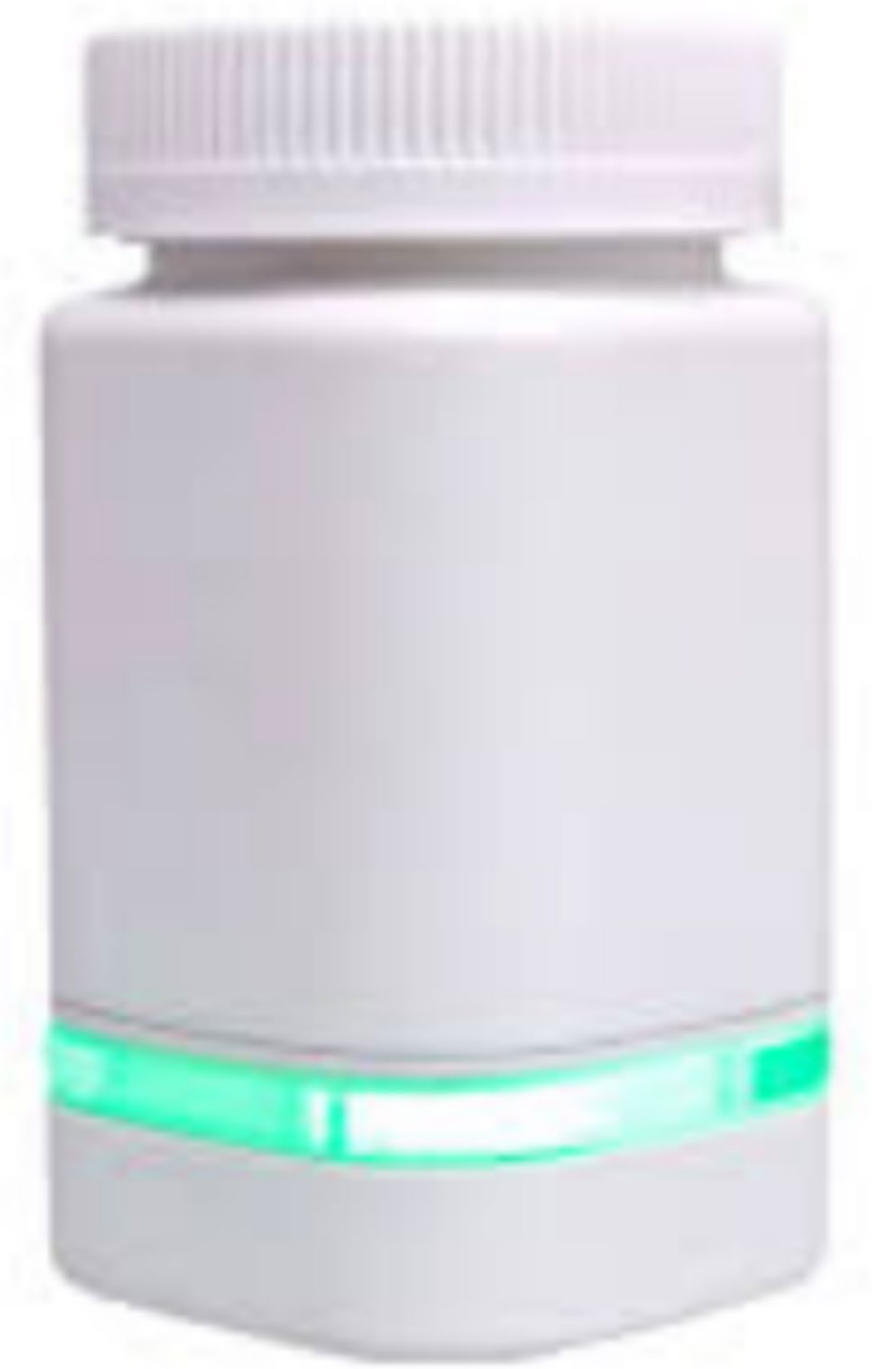
AdhereTech bottle

3) A smart pill bottle: We included an AdhereTech smart pill bottle, HIPAA-compliant, FDA-class I medical devices providing electronic tracking of adherence by measuring the bottle opening (date/time). AdhereTech's bottles automatically collect adherence data in real-time; as participants use the bottles, adherence data (i.e., bottle opening, cellular connectivity, battery power, bottle functioning) is wirelessly sent from the bottles to AdhereTech's servers using sensors and a built-in cellular chip. All data and analytics are available 24/7 on the real-time dashboard. AdhereTech’s bottles also hold a charge for approximately 6 months meaning that participants did not have to remember to charge the bottle during our 6-month study duration. Data related to the bottle’s status was accessible by the study team, thus the study team can remind participants to activate or charge the bottle if necessary. Participants received their bottles with two alerts activated: 1. a glowing LED strip on the bottle that started 1 h before dose time and 2. A single chime when the medication dose was scheduled. See Fig. 3 for the bottle outlook.

### Patient population and recruitment

Eligible patient participants were 18 years or older and had a diagnosis of CML, has been taking TKI ≥ 3 months and not more than 3 years for chronic-phase CML, spoke and read English, and had cellular access and text messaging capability via a mobile phone. Patients were excluded if they had a documented diagnosis of cognitive impairment or psychiatric depression. Potentially eligible patients were reached during their oncology appointments or remotely over the phone and were provided with the opportunity to ask questions and, if not comfortable, to opt out. Once written consent or eConsent was obtained, participants completed a baseline survey and were asked to use the intervention for 6 months. The research staff checked in monthly in person or through a telephone call.

### Measures

Clinical characteristics with regard to diagnosis and medication prescription and duration were extracted from participants'medical records. Pre-intervention and post-intervention assessments involved the completion of various instruments guided by the Necessity and Concerns framework: 1) Average adherence calculated based on daily AdhereTech bottle data was used. 2) The MD Anderson Symptom Inventory (MDASI) is a brief and valid measure of the severity and impact of cancer-related symptoms [24]. 3) The ASK-12 has been used in behavioral trial for assessing perceived barriers to medication adherence [25]. 4) The Beliefs about Medications Questionnaire-specific (BMQ–specific) is a self-report instrument comprising 10 items to elicit an individual's beliefs about their medicines in the domains of necessity of medicines and concerns about medicines. BMQ_diff was calculated by extracting the BMQ concerns score from BMQ necessity [26]. 5) Brief Illness Perception Questionnaire (BIPQ) consists of nine items that assess the cognitive and emotional representations of illness [27]. 6) Medication self-efficacy was measured by the Self-Efficacy for Appropriate Medication Use Scale (SEAMS). 7) The Multidimensional Scale of Perceived Social Support (MSPSS) has been used in behavioral trials for measuring perceived social support among cancer patients [28]. 8) The Impact of Event Scale—Revised (IES-R) was used to measure common posttraumatic stress symptoms and the impact of stressful life events over the past week [29]. 9) To evaluate intervention satisfaction, we employed the Client Satisfaction Quesionnaire-8 item version (CSQ-8), a validated measure that elicits the participant’s perspective on the value of services received [30]. To collect survey responses, participants were provided with a survey link via email or text through the REDCap online platform. Alternatively, paper questionnaires were offered during clinical visits. Participants received compensation of $20, $30, and $40 for each subsequent assessment time points for their time and efforts.

### Post-intervention interviews

Participants were further invited for a post-intervention semi-structured phone interviews to share their experiences and feedback regarding the *txt4 TKI* intervention. The interviews were structured to understand the ease of use, the relevance of self-care messages, any experienced symptoms not asked by the Chatbot’s toxicity assessment, and suggestions for enhancing the intervention.

### Data analysis

Demographic and medical characteristics were summarized with descriptive data. Engagement with the intervention, including the frequency of patient-initiated text responses for additional tips and responses to the Chatbot’s toxicity assessment, was also outlined using descriptive statistics. Paired-*t*-tests were performed to compare 6-month scores with baseline scores for each repeated measure and 95% confidence intervals were presented. Patient adherence data are expressed as a series of daily binary adherence scores then was calculated the percentage od days with correct dosing for a given study period: 0–3 month and 0–6 month. The qualitative data analysis involved identifying themes and categories from the interview transcriptions. A codebook was created based on topical codes from the interviews, enriched with interpretive codes during ongoing analysis. This process ensured organized data grouping into relevant themes. Sample interview quotes illustrating findings were presented.

### Study results

#### Sample characteristics

In total, 30 eligible patients were approached to participate in the pilot study over 8 months and 20 consented to participate. Reasons for declining were either they were not interested (50%) or they were overwhelmed (50%). The sample comprised 60% women, with mean age of 55.3 (SD = 6.5), majority were non-White (65%), not married/no partner (55%), below college education (55%) and with a household income level below $60 K (55%). The majority of participants have been taking their medication for 1–3 years (55%) and 50% were taking Dasatinib, 45% Imatinib, and 5% Bosutinib. Thirty percent of participants had a ECOG score of 1 and 35% reported polypharmacy, currently taking five or more medications, including TKI therapy. Table 2 lists participant characteristics information.

**Table 2:** Baseline characteristics

	**N**	**%**
Age: Mean (SD)	55.3	9.2
African American	7	35
Multi-ethnic	6	30
Female	12	60
Non-married/no partner	11	55
Below college	11	55
Below $60 K	11	55
TKI Medication		
Dasatinib	10	50
Imatinib	9	45
Bosutinib	1	5
Medication duration		
< 1 year	9	45
1–3 years	11	55
ECOG		
0	14	70
1	6	30
Polypharmacy (5 and more medications)	7	35

#### Feasibility

**Fig. 4:**
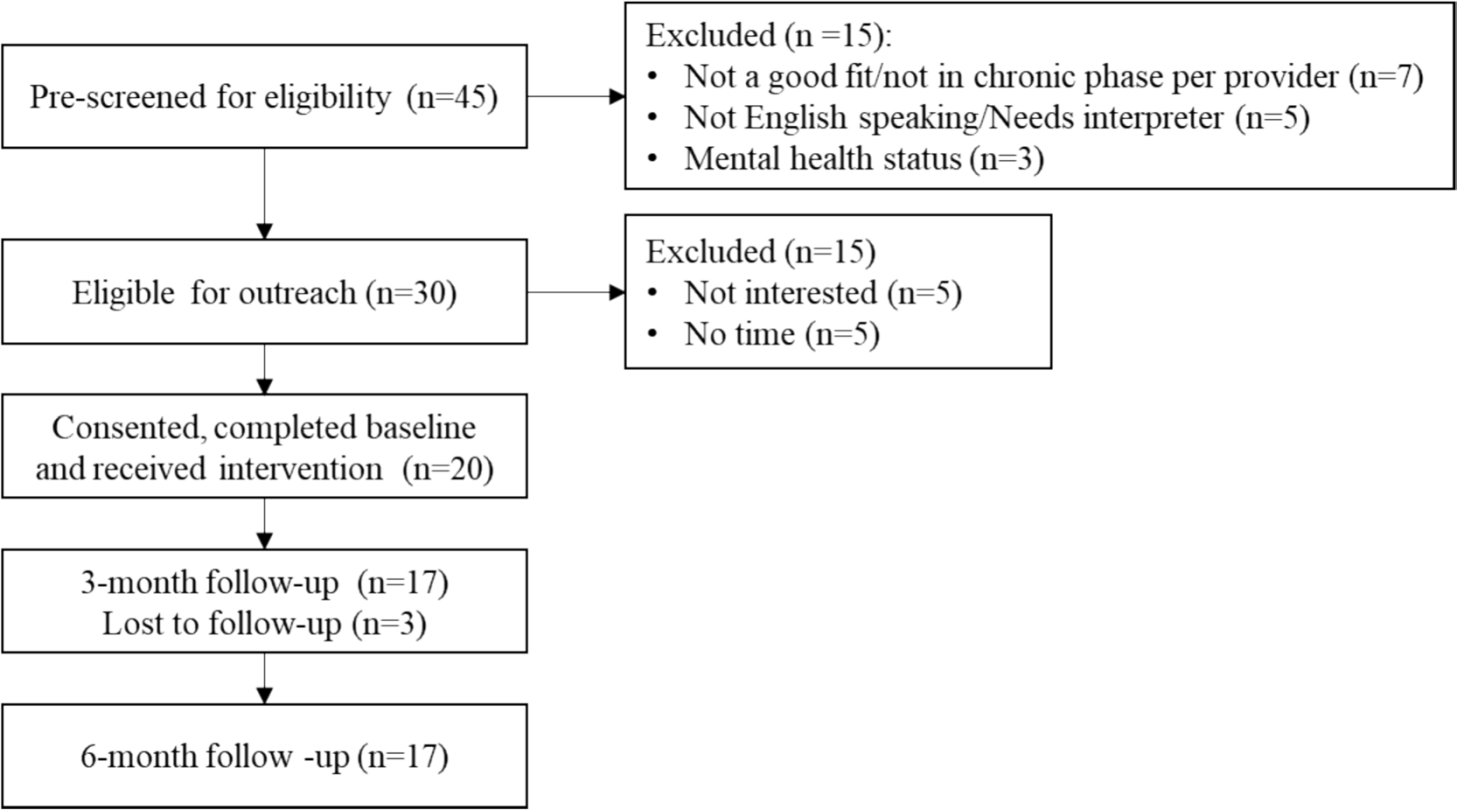
Consort flow diagram

Feasibility was measured through study accrual and attrition rates. Accrual rate over 65% and an attrition rate that does not exceed 30% is feasible. Out of 30 eligible patients approached, 20 consented and enrolled (66.7% consenting rate). At 3-month post-intervention, 17 participants successfully completed the follow-up (15% attrition rate). See Fig. 4. for the patient flow diagram.

#### Acceptability

Acceptability was examined through a satisfaction survey and patient interviews conducted at the 6-month follow-up assessment. Participants reported high perceived utility with the *txt4 TKI* program, with 94.1% rating the program’s quality as “excellent” or “good.” Majority (90.5%) reported they would recommend the intervention to someone facing similar circumstances. The overall satisfaction results based on CSQ-8 indicated a mean score of 3.18 at the 3-month and 3.32 at the 6-month assessment on a scale ranging from 1 (least satisfied) to 4 (extremely satisfied).

Post-intervention phone interview included brief questions covering likes/dislikes, program helpfulness, and recommendations for improvements. Participants appreciated the informative nature of the text messages, finding them helpful in providing information about side effects, medication details, and stress management. Participants valued the functionalities of texting back to the system for more information. The pill bottle, serving as a reminder, received praise for its effectiveness in aiding medication adherence. The emotional support aspect of the program was also acknowledged, with participants finding comfort in knowing support was available when needed.

In terms of future improvements, some participants found certain text messages difficult to understand, and a few indicated a desire for more detailed information. While the pill bottle was praised for its reminder function, a portion of participants found it complicated to use or considered it too large. Additionally, some participants suggested that the intervention could have been more beneficial earlier in their diagnosis and treatment period. See Table 3 for sample post-intervention interview quotes.

**Table 3:** Participant’s interview quotes at 6-month follow-up

	Positive Appraisals	Potential Future Improvement
**Message’s helpfulness**	“it helped me with information about side effects. Messages about nausea and diarrhea was very helpful.”	“Sometimes I find them hard to understand.”
	“it tells me about the medication I was taking it tells me about TKI and things I did not know, very informative.”	“Did not give a great deal of information”
	“the most helpful are messages on different things I could do about stress.”	“I think they would have been most useful earlier in my diagnosis/treatment period but the information was interesting.”
	“Very informative. I like that I could text back to the system.”	
**Pill bottle’s helpfulness**	“reminds me to take my med because sometimes I forgot”	“Bottle is complex to use”
	“The pill bottle, together with a reminder set on my phone worked well together to make sure that I took my pills!”	“I found the pill bottle to be too complicated to use since I couldn’t set it directly and since it was also large”
**Emotional support**	“It was comforting knowing there was support out there if I needed it.”	
	“The program provided useful information about CML and made me feel a sense of shared experience since it referred to the experiences of other CML patients.”	

### Engagement with the txt4 TKI intervention

#### Text messaging interaction

The intervention consists of unprompted messages with topic keywords provided to participants for additional tips or coping strategies. Throughout the 6-month intervention period, 90% of patients engaged by texting back at least once for additional information. Total interaction varied from one message to 49 messages, with an average of 10 messages per participant during the intervention. The most frequently used keywords were “tki” (70.5%), “muscle pain” (31%), and “diarrhea” (28%). Some responses included free-text messages or emojis such as “thumbs up” and “thank you.”

#### Reported symptoms via chatbot

Only five participants (25%) utilized the Chatbot symptom monitoring at least once during the 6-month intervention. Symptoms reported were of moderate severity for nausea (80%) and muscle pain (65%). Reasons for not responding included the absence of symptoms at the time, feeling burdened, and forgetfulness.

#### Change in adherence and psychosocial measures from baseline to 6-month follow-up

Descriptive data and comparisons between baseline, 3-month, and 6-month follow-up time points for symptom severity, psychosocial measures and adherence rates are detailed in Table 4. Paired sample *t*-tests with confidence intervals indicate that the only statistically significant change from baseline to 6 months was an increase in medication self-efficacy, measured by SEAMS. Overall, this cohort of CML patients reported relatively lower symptom severity (MDASI), perceived fewer barriers to adherence (ASK-12), a stronger belief in the necessity of TKI over concerns (BMQ_Diff), and a moderately negative perception about their condition (BIPQ). Furthermore, they reported moderately higher self-efficacy in medication management (SEAMS) and perceived receiving good social support (MSPSS). Perceived stress levels, as measured by IES-R, did not reach a clinical level. The mean adherence rate to TKI measured by smart pill bottles was 88.85% during the first three months and 78.76% from months 3 to 6.

**Table 4:** Changes in measures from baseline to follow-up

Measure (Range)	Baseline N = 20 Mean (SD)	3 Month N = 17 Mean (SD)	6 Month N = 17 Mean (SD)	6 Month Change Paired Difference (95% C.I.)	*P* (paired *t* test)
MDASI (0- 10)	2.74 (2.21)	3.01 (2.32)	3.41 (2.55)	0.62 (−0.54, 1.78)	0.27
ASK-12 (0–60)	15.00 (7.18)	12.88 (7.43)	13.00 (7.79)	−2.00 (−4.91 ~ 0.91)	0.16
BMQ_Diff (−25–25)	5.47 (7.07)	6.7 (6.51)	6.07 ± 6.54	0.57 (−2.76–3.91)	0.68
BIPQ (0–80)	39.05 (9.56)	36.59 (9.73)	36.75 (9.91)	−4.00 (−10.01, 2.01)	0.18
SEAMS (3–39)	28.83 (5.74)	30.13 (5.15)	34.64 (4.38)	4.43 (1.26, 7.56)	< 0.001
MSPSS (1–84)	66.93 (12.59)	65.50 (19.44)	65.25 (17.48)	−1.69 (−5.51, 2.14)	0.36
IES-R (0–88)	17.33 (17.24)	17.47 (20.50)	15.60 (18.98)	−1.73 (−6.06, 2.59)	0.40
Overall adherence rate: 0–3 month and 0–6 month		88.85%	82.80%	−0.06 (−0.02, 0.10)	0.11

## Discussion

This study demonstrated the feasibility and acceptability of an interactive *txt4 TKI* intervention tailored for individuals with CML undergoing TKI therapy. While TKI treatment is highly effective for CML patients, the associated side effects and the long-term commitment to daily pills have presented challenges to adherence in this population. Our initiative aims to bridge the care gap that often emerges during medication use, a gap accentuated by disruptions such as those caused by the COVID-19 pandemic.

Despite almost two decades since the introduction of the first TKI, studies on approaches to improve adherence to TKI therapy for CML patients are still limited. Existing research lacks patient-focused interventions, especially those utilizing mobile digital approaches that have shown potential in enhancing oral cancer medication adherence in other contexts [31]. To fill the void and address unmet patient needs, during the program's development in our study, findings from phase 1 interviews informed the design of our *txt4 TKI*—ranging from symptom management to emotional support and information acquisition, addressing the necessity and concerns of the TKI therapy. The intervention’s dual TXT and Chatbot interfaces facilitate patient interactions, fostering engagement and empowering patients to manage their well-being more easily, supplemented by a smart pill bottle serving as a cue for action.

Participants at the 6-month follow-up reported positive assessment of the *txt4 TKI* intervention. The text messages were praised for their helpfulness, offering valuable information about side effects, medication details, and stress management and the pill bottle emerged as a useful tool, reminding participants to take their medication. Emotional support was highlighted, with participants finding comfort in the availability of support and shared experiences through the program. Although the impact of the intervention, based on pre-and post-test measures, was modest overall, a significant improvement was observed in patient’s perceived medication self-efficacy. Similar to our findings, other studies have also reported significant improvement in self-efficacy resulting from adherence-promotion interventions for cancer patients using oral anticancer drugs [32, 33]. Our intervention target keys determinants of patient behaviors through facilitating patient monitoring, allowing side effects to be self-identified early, and providing real-time feedback for patients, thereby, providing patients with a sense of control, an important aspect of treatment adherence self-efficacy [34].

However, despite the generally qualitative positive feedback and increased self-efficacy reported by participants, the objective adherence rate did not fully align with these positivities. Although the decline in adherence from 3 to 6 months was not statistically significant and remained above 80% overall, a common benchmark for oral medication adherence [35], several factors may explain the decline. One potential reason is the absence of baseline adherence data or a control group for comparison. Second, adherence may have improved initially due to the introduction of a new supportive intervention, while compliance declining over time because the intervention remained static, not addressing maintenance barriers. This pattern aligns with findings from other proof-of-concept studies, including a smartphone-based adherence program for cancer patients that also used an electronic bottle for objective adherence tracking [36]. Similar to our study, that work noted greater utility for patients new to treatment, challenges with bottle usability, high acceptability, and a decline in adherence over time [36]. Given these challenges, future intervention designs should prioritize user-friendly technology with simpler, more flexible features to better integrate into patients’ daily routines that support sustained long-term adherence. As smart pill bottle technology continues to advance, future designs are likely to become more user-friendly and cost-effective, improving their practicality for routine use. While the current version may present challenges, particularly for patients needing to transport large bottles, our feasibility findings still pave the way for future dissemination of this type of smart bottle in non-research settings. Additionally, future iterations of the intervention could explore transitioning patients to more practical and scalable reminder methods—such as phone alarms or app-based notifications—once consistent medication-taking habits are established. This approach may enhance long-term feasibility and support broader integration into routine oncology care.

Given that long-term use of TKI allows patients to manage CML at home, improving patient’s self-management with medication is critical. Regular and consistent dosing is essential to achieve optimal molecular response outcomes and eventually allow for treatment discontinuation [37]. Thus, it is crucial to evaluate tailored adherence-promotion interventions that facilitate sustainable, long-term adherence to ensure continued success in TKI therapy. Our study participant’s feedback from post-intervention interviews suggests that this type of intervention might be particularly beneficial for individuals initiating TKI therapy. On the contrary. although it did not use a text messaging approach, a randomized controlled trial of a smartphone app aimed at improving adherence to oral cancer therapy found no overall effect [38]. However, the app did improve adherence among patients with higher anxiety or lower baseline adherence [38]. Combined with our findings, these results suggest the potential value of tailoring interventions to individual characteristics. Future research should explore the specific needs and preferences of patients at different stages of TKI therapy or with varying risk factors to better personalize adherence support.

### Study limitations

We acknowledge limitations in our study design and execution. The pilot nature of the study, reflected in the small sample size, may impact the generalizability of findings, as it may not fully capture patient diversity in demographics, socioeconomic status, and treatment settings. Biases inherent to a single-arm pilot design and the lack of adherence baseline assessment also influence outcome interpretation. Lack of a control group precluded efficacy evaluation, a goal for future RCTs. Future directions also need to consider involving EMR integration for direct information exchange and communication between patients and providers. Post-intervention feedback revealed reduced intervention usage due to reasons like symptom-free status or the complexity of the pill bottle or the chatbot interface. Further, our chatbot's structured approach limits its understanding of patient contexts. Future research may explore integrating AI to enhance responsiveness to diverse patient expressions.

## Conclusion

Text-based with a smart pill bottle interventions are feasible in patients with CML prescribed for TKI for symptom management and medication adherence, has potential in improving patient’s self-efficacy and engaging behavior changes. Our intervention, theoretically developed with patient-inputs, presents an easy to use and readily accessible solution, that will need to be further enhanced and evaluated in larger clinical trials.

## Data Availability

No datasets were generated or analysed during the current study.

## References

[1] Hehlmann, R., M. Lauseker, S. Jung-Munkwitz, A. Leitner, M.C. Müller, N. Pletsch, . . . L. Balleisen, Tolerability-adapted imatinib 800 mg/d versus 400 mg/d versus 400 mg/d plus interferon-a in newly diagnosed chronic myeloid leukemia. J Clin Oncol, 2011. 29(12): p. 1634–1642. 10.1200/JCO.2010.32.0598 21422420

[2] Howlader, N., A. Noone, M. Krapcho, J. Garshell, D. Miller, S. Altekruse, . . . Z. Tatalovich, SEER cancer statistics review, 1975–2012. Bethesda, MD: National Cancer Institute, 2015. 2015.

[3] Noens L, Hensen M, Kucmin-Bemelmans I, Lofgren C, Gilloteau I, Vrijens B (2014) Measurement of adherence to BCR-ABL inhibitor therapy in chronic myeloid leukemia: current situation and future challenges. Haematologica 99(3):437–447 24598855 10.3324/haematol.2012.082511 PMC3943306

[4] Efficace, F., M. Baccarani, G. Rosti, F. Cottone, F. Castagnetti, M. Breccia, . . . F. Mandelli, Investigating factors associated with adherence behaviour in patients with chronic myeloid leukemia: an observational patient-centered outcome study. Br J Cancer, 2012. 107(6): p. 904–9. 10.1038/bjc.2012.348 PMC3464760 22871884

[5] Noens, L., M.-A. Van Lierde, R. De Bock, G. Verhoef, P. Zachée, Z. Berneman, . . . K. MacDonald, Prevalence, determinants, and outcomes of nonadherence to imatinib therapy in patients with chronic myeloid leukemia: the ADAGIO study. Blood, 2009. 113(22): p. 5401–5411. 10.1182/blood-2008-12-196543 19349618

[6] Kekäle M, Talvensaari K, Koskenvesa P, Porkka K, Airaksinen M (2014) Chronic myeloid leukemia patients’ adherence to peroral tyrosine kinase inhibitors compared with adherence as estimated by their physicians. Patient Prefer Adherence 8:1619 25473270 10.2147/PPA.S70712 PMC4246993

[7] Darkow, T., H.J. Henk, S.K. Thomas, W. Feng, J.-F. Baladi, G.A. Goldberg, . . . J. Cortes, Treatment interruptions and non-adherence with imatinib and associated healthcare costs. Pharmacoeconomics, 2007. 25(6): p. 481–496. 10.2165/00019053-200725060-00004 17523753

[8] Kapoor, J., N. Agrawal, R. Ahmed, S.K. Sharma, A. Gupta, and D. Bhurani, Factors influencing adherence to imatinib in Indian chronic myeloid leukemia patients: a cross-sectional study. Mediterranean journal of hematology and infectious diseases, 2015. 7(1). 10.4084/MJHID.2015.013 PMC4344173 25745540

[9] Marin, D., A. Bazeos, F.-X. Mahon, L. Eliasson, D. Milojkovic, M. Bua, . . . K. Kozlowski, Adherence is the critical factor for achieving molecular responses in patients with chronic myeloid leukemia who achieve complete cytogenetic responses on imatinib. Journal of clinical oncology: official journal of the American Society of Clinical Oncology, 2010. 28(14): p. 2381–2388. 10.1200/JCO.2009.26.3087 PMC6366340 20385986

[10] Deedwania, P.C., M. Gupta, M. Stein, J. Yčas, A. Gold, and I.S. Group, Comparison of rosuvastatin versus atorvastatin in South-Asian patients at risk of coronary heart disease (from the IRIS Trial). The American journal of cardiology, 2007. 99(11): p. 1538-1543 10.1016/j.amjcard.2007.01.028 17531577

[11] Bauer S, Buchanan S, Ryan I (2016) Tyrosine Kinase Inhibitors for the Treatment of Chronic-Phase Chronic Myeloid Leukemia: Long-Term Patient Care and Management. J Adv Pract Oncol 7(1):42–54 27713843 10.6004/jadpro.2016.7.1.3 PMC5045277

[12] Geissler, J., G. Sharf, F. Bombaci, M. Daban, J. De Jong, T. Gavin, . . . V.S. Hoffmann, Factors influencing adherence in CML and ways to improvement: Results of a patient-driven survey of 2546 patients in 63 countries. J Cancer Res Clin Oncol, 2017. 143(7): p. 1167–1176. 10.1007/s00432-017-2372-z PMC11819081 28289895

[13] Tan, B.K., P.C. Bee, S.S. Chua, and L.-C. Chen, Monitoring and improving adherence to tyrosine kinase inhibitors in patients with chronic myeloid leukemia: a systematic review. Patient preference and adherence, 2021: p. 2563–2575. 10.2147/PPA.S269355 PMC8608409 34819724

[14] Wen, K.-Y., L.J. Goldstein, R. Smith, and S.M. Miller, Mobile text messaging to improve adjuvant hormone therapy and side effect management among breast cancer patients: A pilot RCT. 2019, American Society of Clinical Oncology.

[15] Tan, E.H., A.L.A. Wong, C.C. Tan, P. Wong, S.H. Tan, L.E.Y. Ang, . . . S.C. Lee, Improving medication adherence with adjuvant aromatase inhibitor in women with breast cancer: A randomised controlled trial to evaluate the effect of short message service (SMS) reminder. The Breast, 2020. 53: p. 77–84. 10.1016/j.breast.2020.06.012 PMC7375684 32652462

[16] Piau A, Crissey R, Brechemier D, Balardy L, Nourhashemi F (2019) A smartphone Chatbot application to optimize monitoring of older patients with cancer. Int J Med Informatics 128:18–23 10.1016/j.ijmedinf.2019.05.013 31160007

[17] Chen J, Ou L, Hollis SJ (2013) A systematic review of the impact of routine collection of patient reported outcome measures on patients, providers and health organisations in an oncologic setting. BMC Health Serv Res 13:211 23758898 10.1186/1472-6963-13-211 PMC3700832

[18] Basch, E., A.M. Deal, M.G. Kris, H.I. Scher, C.A. Hudis, P. Sabbatini, . . . D. Schrag, Symptom Monitoring With Patient-Reported Outcomes During Routine Cancer Treatment: A Randomized Controlled Trial. Journal of clinical oncology : official journal of the American Society of Clinical Oncology, 2016. 34(6): p. 557–565. 10.1200/JCO.2015.63.0830 PMC4872028 26644527

[19] Beck SL, Eaton LH, Echeverria C, Mooney KH (2017) SymptomCare@Home: Developing an Integrated Symptom Monitoring and Management System for Outpatients Receiving Chemotherapy. Comput Inform Nurs 35(10):520–529 28570285 10.1097/CIN.0000000000000364 PMC5628091

[20] Pereira-Salgado, A., J.A. Westwood, L. Russell, A. Ugalde, B. Ortlepp, J.F. Seymour, . . . P. Schofield, Mobile Health Intervention to Increase Oral Cancer Therapy Adherence in Patients With Chronic Myeloid Leukemia (The REMIND System): Clinical Feasibility and Acceptability Assessment. JMIR Mhealth Uhealth, 2017. 5(12): p. e184. 10.2196/mhealth.8349 PMC5738545 29212628

[21] Ellsworth, G.B., L.A. Burke, M.T. Wells, S. Mishra, M. Caffrey, D. Liddle, . . . R.M. Gulick, Randomized Pilot Study of an Advanced Smart-Pill Bottle as an Adherence Intervention in Patients With HIV on Antiretroviral Treatment. J Acquir Immune Defic Syndr, 2021. 86(1): p. 73–80. 10.1097/QAI.0000000000002519 PMC7735215 33306564

[22] Sohn A, Speier W, Lan E, Aoki K, Fonarow GC, Ong MK, Arnold CW (2020) Integrating remote monitoring into heart failure patients’ care regimen: A pilot study. PLoS ONE 15(11):e0242210 33211733 10.1371/journal.pone.0242210 PMC7676713

[23] Clifford S, Barber N, Horne R (2008) Understanding different beliefs held by adherers, unintentional nonadherers, and intentional nonadherers: application of the necessity–concerns framework. J Psychosom Res 64(1):41–46 18157998 10.1016/j.jpsychores.2007.05.004

[24] Cleeland, C.S., T.R. Mendoza, X.S. Wang, C. Chou, M.T. Harle, M. Morrissey, and M.C. Engstrom, Assessing symptom distress in cancer patients: the MD Anderson Symptom Inventory. Cancer: Interdisciplinary International Journal of the American Cancer Society, 2000. 89(7): p. 1634–1646. 10.1002/1097-0142(20001001)89:7<1634::aid-cncr29>3.0.co;2-v 11013380

[25] Matza LS, Park J, Coyne KS, Skinner EP, Malley KG, Wolever RQ (2009) Derivation and validation of the ASK-12 adherence barrier survey. Ann Pharmacother 43(10):1621–1630 19776298 10.1345/aph.1M174

[26] Horne R, Weinman J, Hankins M (1999) The beliefs about medicines questionnaire: the development and evaluation of a new method for assessing the cognitive representation of medication. Psychol Health 14(1):1–24

[27] Broadbent E, Petrie KJ, Main J, Weinman J (2006) The brief illness perception questionnaire. J Psychosom Res 60(6):631–637 16731240 10.1016/j.jpsychores.2005.10.020

[28] Zimet GD, Dahlem NW, Zimet SG, Farley GK (1988) The multidimensional scale of perceived social support. J Pers Assess 52(1):30–41 10.1080/00223891.1990.9674095 2280326

[29] Creamer M, Bell R, Failla S (2003) Psychometric properties of the impact of event scale—revised. Behav Res Ther 41(12):1489–1496 14705607 10.1016/j.brat.2003.07.010

[30] Attkisson CC, Zwick R (1982) The Client Satisfaction Questionnaire: Psychometric properties and correlations with service utilization and psychotherapy outcome. Eval Program Plann 5(3):233–237 10259963 10.1016/0149-7189(82)90074-x

[31] Ross XS, Gunn KM, Patterson P, Olver I (2018) Mobile-based oral chemotherapy adherence–enhancing interventions: Scoping review. JMIR Mhealth Uhealth 6(12):e11724 30578182 10.2196/11724 PMC6320412

[32] Spoelstra, S.L., A. Sikorskii, A. Majumder, P.S. Burhenn, M. Schueller, and B. Given, Oral Anticancer Agents. Clinical Journal of Oncology Nursing, 2017. 21(2). 10.1188/17.CJON.157-160 28315545

[33] Spoelstra, S.L., C.W. Given, A. Sikorskii, C.K. Coursaris, A. Majumder, T. DeKoekkoek, . . . B.A. Given. Feasibility of a text messaging intervention to promote self-management for patients prescribed oral anticancer agents. in Oncol Nurs Forum. 2015. 10.1188/15.ONF.647-657 26488833

[34] Náfrádi L, Nakamoto K, Schulz PJ (2017) Is patient empowerment the key to promote adherence? A systematic review of the relationship between self-efficacy, health locus of control and medication adherence. PLoS ONE 12(10):e0186458 29040335 10.1371/journal.pone.0186458 PMC5645121

[35] Peterson AM, Nau DP, Cramer JA, Benner J, Gwadry-Sridhar F, Nichol M (2007) A checklist for medication compliance and persistence studies using retrospective databases. Value in health 10(1):3–12 17261111 10.1111/j.1524-4733.2006.00139.x

[36] Skrabal Ross, X., K.M. Gunn, V. Suppiah, P. Patterson, T. Boyle, C. Carrington, . . . I. Olver, A smartphone program to support adherence to oral chemotherapy in people with cancer: Proof-of-concept trial. Asia Pac J Clin Oncol, 2022. 18(5): p. e378-e387. 10.1111/ajco.13656 35098675

[37] Ross DM, Hughes TP (2014) How I determine if and when to recommend stopping tyrosine kinase inhibitor treatment for chronic myeloid leukaemia. Br J Haematol 166(1):3–11 24754670 10.1111/bjh.12892

[38] Greer, J.A., J.M. Jacobs, N. Pensak, L.E. Nisotel, J.N. Fishbein, J.J. MacDonald, . . . J.S. Temel, Randomized Trial of a Smartphone Mobile App to Improve Symptoms and Adherence to Oral Therapy for Cancer. J Natl Compr Canc Netw, 2020. 18(2): p. 133–141. 10.6004/jnccn.2019.7354 32023526

